# Vitamin E Loaded Naringenin Nanoemulsion via Intranasal Delivery for the Management of Oxidative Stress in a 6-OHDA Parkinson's Disease Model

**DOI:** 10.1155/2019/2382563

**Published:** 2019-04-14

**Authors:** Bharti Gaba, Tahira Khan, Md Faheem Haider, Tausif Alam, Sanjula Baboota, Suhel Parvez, Javed Ali

**Affiliations:** ^1^Department of Pharmaceutics, School of Pharmaceutical Education and Research, Jamia Hamdard, New Delhi 110062, India; ^2^Department of Pharmacology, School of Pharmaceutical Education and Research, Jamia Hamdard, New Delhi 110062, India; ^3^Department of Toxicology, School of Chemical and Life Sciences, Jamia Hamdard, New Delhi 110062, India

## Abstract

**Purpose:**

The present study is an attempt to develop a vitamin E loaded naringenin (NRG) Nanoemulsion (NE) for direct nose-to-brain delivery for better management of Parkinson's disease (PD).

**Methods:**

The optimized NE was evaluated for efficacy in PD using multiple behavioral studies (including narrow beam test, muscular coordination test, grip strength test, forced swimming test, and akinesia test) in a rat model. Optimized formulation was evaluated for droplet size, polydispersity index (PDI), refractive index, transmittance, zeta potential, and viscosity.

**Results:**

Optimized NE had a droplet size of 38.70 ± 3.11nm, PDI of 0.14 ± 0.0024, refractive index of 1.43 ± 0.01, transmittance of 98.12 ± 0.07 %, zeta potential of − 27.4 ± 0.14 mV, and viscosity of 19.67 ± 0.25 Pa s. Behavioral studies showed that 6-OHDA induced PD in rats were successfully reversed when administered with NRG NE intranasally along with the levodopa. While the levels of GSH and SOD were significantly higher, levels of MDA were significantly lower in the group treated with NRG NE via intranasal route along with levodopa.

**Conclusion:**

Encouraging results from current study provide evidence for possible efficacy of a novel noninvasive intranasal delivery system of NRG for management of PD related symptoms.

## 1. Introduction

Approximately 6.3 million people are affected from Parkinson's disease (PD) as suggested by World Health Organization. In 2016, 6.1 million individuals worldwide had Parkinson's disease, of whom 2.9 million (47.5%) were women and 3.2 million (52.5%) were men. The number of individuals with Parkinson's disease in 2016 was 2.4 times higher than in 1990 (2.5 million, 95%). This increase was not solely due to the increasing number of older people because global age-standardized prevalence rates increased by 21.7% from 1990 to 2016 compared with an increase of 74.3% for crude prevalence rates. The increase in age-standardized prevalence rates between 1990 and 2016 was similar in men (21.4%) and women (19.3%). Age-standardized prevalence rates of Parkinson's disease by country varied greater than five times, with the highest rates generally in high-income North America and lowest rates in sub-Saharan Africa [1]. Among the other neurodegenerative diseases, PD is ranked as second most prevalent disorder across the world. It generally affects the middle age or elderly population and is characterized by symptoms related to dopamine deficiency such as resting tremors, muscular rigidity, bradykinesia, postural instability, and disturbance in gait [2, 3]. There are various different endogenous and exogenous reasons accountable for this disease including oxidative stress, heredity, mitochondrial dysfunction, aging, inflammation, and various neurotoxins. Oxidative stress (OS) emerges to be the leading cause of PD. More specifically dopamine is metabolized due to oxidative stress into hydrogen peroxide and other reactive oxygen species (ROS). The treatment available for PD therefore usually aims to correct dopamine deficiency. For the treatment of PD, levodopa has been widely used as the first line treatment owing to its potential of controlling the motor dysfunction symptoms leading to the successful crossing of blood–brain barrier (BBB). However, using levodopa for the long-term may lead to various motor deficits including disturbances with balance, gait, speech, and posture [4].

Fruits and vegetables typically contain flavonoids, which are the nonnutrients, present in addition to vital vitamins and nutrients present in them. Chemically flavonoids are a collection of polyphenolic compounds distinguished via a benzene-g-pyrone structure. Approximately 4000 flavonoids species have been described which are further categorized into flavones, isoflavanones, anthocyanidins, flavonols, catechins, and flavanones [5–7]. Recently flavonoids have gained attention due to their anticancer, antioxidant, antiviral, and anti-inflammatory properties. One of the citrus flavanone naringenin (NRG) (4',5,7-trihydroxyflavanone) occurs copiously in citrus fruits such as grapes, grapefruit, blood orange, lemons, pummelo, and tangerines [8]. NRG has been reported to display variety of effects on the biological system including like anti-inflammatory, antioxidant, antifibrogenic, antiatherogenic, and anticancer. The advantageous effect of NRG is basically due its antioxidant activity. It has the ability to scavenge oxygen-free radicals and chelate metals that inhibit enzymes to thwart oxidation of low-density lipoproteins. However NRG has some limitations including its poor bioavailability and water insolubility [9, 10].

Due to several challenges with use of existing dopaminergic medications for PD, various new formulations have been envisaged for drug delivery directly to the brain through olfactory and trigeminal neuronal pathways instead. There are many invasive and noninvasive techniques that may be used for the brain delivery, out of which intranasal route of drug delivery which is noninvasive and delivery is directly to the brain has been extensively scrutinized. Delivery of lipophilic drug directly to brain in the form of Nanoemulsion (NE) via intranasal route has been found to be one of the best possible approach [11, 12]. In addition, solubility of the lipophilic drugs is also enhanced which ultimately improves its permeation across mucosa [13]. NEs are dispersions of two immiscible phases; it may be transparent or translucent having nanometric size ranging between 20 and 200 nm. On the whole, NEs are kinetically stabilized isotropic systems which by using surfactant or mix of surfactant along with a cosurfactant immiscible liquid are made miscible to form a single phase [14, 15]. In addition, they possess the advantage over macroemulsion of having free energy and elevated surface area; also it is stabilized against creaming, sedimentation, coalescence, and flocculation [13]. NEs can be formulated with low quantities of emulsifier having approximately 4–10 wt%, hence minimizing surfactant related toxicity problems [16].

Intranasal drug delivery has several advantages over other route of deliveries due to the presence of direct connection between the nasal cavity and central nervous system (CNS). Intranasal route has been extensively studied as an unconventional, convenient, and noninvasive route to deliver the lipophilic drug directly to the brain bypassing the BBB and ultimately enhancing the concentration of drug in the CNS [17, 18]. This route is believed to be highly vascularized and permeable routes having a large surface area and high total blood flow per cm^3^. In addition, quick onset of therapeutic action is created by the rapid absorption of drug due to the low levels of enzyme [19]. In addition, as the drug's systemic exposure is reduced by using the intranasal route it directly dwindles the systemic side effects. Limitations of this route are the delivery of an explicit quantity of formulation, which is 25–200 *μ*l, poor nasal permeability, and rapid mucociliary clearance. These problems can be overcome by the addition of chemical penetration enhancers or colloidal drug delivery systems may be used namely nanoparticles, liposomes and NEs. Based on the existing evidence regarding PD related symptoms caused due to oxidative stress, the delivery of an effective antioxidant via intranasal route will result in higher therapeutic concentration of antioxidant in brain, thereby reducing PD symptoms.

From the previous studies, the effect of vitamin E in conjunction with an additional antioxidant was established for neurodegenerative disorder like Alzheimer's disease [20]. Also, intranasal route for the effective brain delivery was also investigated and proven to be beneficial [20, 21]. Hence, main aim of the present work is to develop a vitamin E loaded NRG NE to be given via intranasal route for the direct and effective brain delivery for PD. In the addition, the current study also examined the synergistic effect of vitamin E and NRG in* in vivo* behavioral studies when administered intranasally on behavioral dysfunction in a 6-OHDA induced rat model of PD. The delivery of naringenin to brain by NRG NE was also determined by estimating NRG levels in brain homogenate and estimation of various pharmacokinetic parameters.

## 2. Materials and Methods

### 2.1. Materials

NRG and 6-OHDA were purchased from Sigma Aldrich (St. Louis, MO, USA). Oils such as peanut oil and olive oil were purchased from Loba Chemie (Mumbai, India). From Gattefosse (Saint Priest, Cedex, France) other oils such as capryol 90, Labrafac, capmul, peceol, Polyethylene glycol 400, Transcutol-HP, Captex 200-P, and Sorbitan sesquioleate were procured. From Hi-media laboratories Pvt. Ltd. (Mumbai, India) dialysis membrane-70 (molecular weight cut-off: 12,000−14,000 Da), LA 393-1 MT was purchased. Tween 80, Castor oil, and HPLC grade methanol were procured from Merck (Schuchardh, Hokenbrunn, Germany). Vitamin E was procured from Evion Capsules available in market. Water and methanol were bought from Fischer Scientific Co. (Mumbai, India). Every chemical and solvent used throughout the study were of analytical grade.

### 2.2. Methods 

#### 2.2.1. Method of Analysis by HPLC

NRG was assayed by HPLC using a system consisting of a quaternary LC-10AT VP pump equipped with UV/VIS detector, SPD-10AVP column oven (Shimadzu), a Rheodyne injector, and chromatography data system software (CLASS-VP Ver 6.14 SP1). Chromatographic separation was carried out on column (LiChrospher®100 RP-18 (5 *μ*m), Merck, Darmstadt, Germany) with a mobile phase consisting of a 7:3 ratio of methanol and water with 0.1 % glacial acetic acid at ambient temperature (25 ± 0.5°C) having a flow rate of 1 ml/min. Samples (20 *μ*l) were filtered through microfilters having 0.45 *μ*m pore size and Rheodyne injector was used for injecting the samples which were then examined at 289 nm [22].

#### 2.2.2. Excipients Selection

Excipients were selected based on their solubility and miscibility with the oils; basically the drug should exhibit higher solubility in the oil phase for accommodating the drug in the solubilized form prohibiting it to get precipitated which finally leads to instability of NE's [23, 24]. Excess of NRG was taken in vials (5 ml size) with simultaneous addition of 2 ml of different oils (namely, soy bean oil, almond oil, olive oil, vitamin E, grape seed oil, rice bran oil, and linseed oil), 2 ml of different surfactants (Tween 20, Tween 60, Tween 80, Solutol HS 15, and Labrasol® (medium chain triglyceride)), and 2 ml of different cosurfactants (Transcutol-HP, lauroglycol 90, propylene glycol, Plurol Oleique, and PEG 400). The vials were placed under continuous shaking using vortex mixer (Nirmal International, Delhi, India) at 25 ± 2°C for 24 h. The vials containing the resultant mixtures were then centrifuged at 4500 rpm for 20 min to separate the insoluble drug. The obtained supernatant was filtered for further investigation for NRG content by means of validated HPLC method (*λ*_max_ = 289 nm). Miscibility studies were also carried out by adding selected oil to surfactant or cosurfactant in 1:1 ratio. The mixture was further vortexed for about 15 min [12, 23]. The mixtures so obtained were allowed to stand for 24 h at room temperature which were further observed for any color change, sign of turbidity, or phase separation.

#### 2.2.3. Pseudoternary Phase Diagram Construction

Pseudoternary phase diagrams were constructed to identify the maximum amount of NE formation. Aqueous phase titration method was used to formulate the NE. Phase diagrams were constructed using vitamin E: Capryol 90 (1:1) as the oil, Tween 80 as the surfactant, Transcutol-HP as the cosurfactant, and distilled water as the aqueous phase. Different volume ratios (1:1, 1:2, 2:1, 3:1, 4:1, and 5:1) of surfactant and cosurfactant (S_mix_) were combined to attain different pseudoternary phase diagrams. For preparing each phase diagram, 16 different volume ratios of oil and S_mix_ (1:9, 1:8, 1:7, 1:6, 1:5, 2:8 (1:4), 1:3.5 (2:7), 1:3 (2:6), 3:7 (1:2.3), 1:2, 4:6 (1:1.5), 5:5 (1:1), 6:4 (1:0.7), 7:3 (1:0.43), 8:2 (1:0.25), and 9:1 (1:0.1)) were vortexed to obtain a clear and homogenous system, following the slow aqueous phase titration using micropipette under continuous stirring. After successful addition of aqueous phase to each vial, they were examined for physical state by visible observation. They were classified as (a) NE when the mixture appeared to be transparent and easily flowable, (b) emulsion when the mixture appears to be cloudy or milky or phase-separated, (c) nanogel when the mixture forms a transparent gel, and (d) emollient gel, when the mixture turns milky and nonflowable even inclining on 90°. CHEMIX School software version 4.0 (Arne Standnes, USA) was used for the creating phase diagrams. NE regions were recognized on a pseudoternary phase diagram that have one axis representing the aqueous phase, second demonstrating the oily phase and the third one representing the S_mix_ in a fixed mass ratio [16].

### 2.3. Preparation of Drug Loaded NE

Aqueous titration method was used for formulating the drug loaded NE. Using vortex mixer (Nirmal International, Delhi, India), a predetermined quantity of NRG was made to dissolved in oily phase, then a fixed amount of S_mix_ was added while continuously stirring the mix using magnetic stirrer (Remi Instrument Ltd., Mumbai, India). Distilled water in specific quantity was then added to the mixture drop wise along with continuous stirring till a transparent and homogeneous NE is formulated [24].

### 2.4. Transmittance (%T)

Formulated NE was measured for percentage transmittance (%T) by means of UV spectrophotometer (Shimadzu Corp, Kyoto, Japan) at 650 nm alongside blank as distilled water [23, 24].

### 2.5. Physical Stability Testing of NEs

#### 2.5.1. Heating-Cooling Cycle

NRG NE was exposed to varied temperatures (4°C and 45°C for not more than 48 h) to determine the consequence of temperature variations on its stability. All measurements were done in triplicate [25, 26].

#### 2.5.2. Centrifugation Study

To detect any creaming, cracking or phase separation NRG NE was centrifuged for 30 mins at 5000 rpm. All measurements were done in triplicate [25, 26].

#### 2.5.3. Freeze Thaw Cycle

NRG NE was exposed to 3 freeze thaw cycles ranging between −21°C and +25°C with storage at each temperature for not less than 48 h to observe the efficiency of dispersibility [25, 26].

### 2.6. Refractive Index and Viscosity

Few drops of the NRG NE were placed on the slide to measure the refractive index (RI) using an Abbe refractometer, which is standardized using castor oil. All measurements were done in triplicate. Brookfield DV III ultra V6.0 RV cone and plate viscometer (Brookfield Engineering Laboratories, Inc., Middleboro, MA) was used to measure the viscosity of the NRG NE without dilution. All of the measurements were carried out using Rheocalc V2.6 software at 25 ± 0.5°C temperature [12].

### 2.7. Globule Size and Surface Charge Analysis

Globule size, zeta potential and polydispersity index (PDI) of NE were determined by using a zetasizer (Nano-ZS90, Malvern Instruments, Worcestershire, UK). Before each measurement, all the formulations were diluted using deionized distilled water for about 200 times. These were then subjected to vigorous shaking for minimizing the multiple scattering effects. 1 ml aliquot from this mixture was pipette out which was then added on the sample cell for the measurement of average globule diameter. Light scattering was measured at 25°C at 90° angle. Afterwards, 1 ml of each sample was taken into clear polystyrene cuvettes independently for the determination of globule size. All measurements were then done in triplicate [27, 28].

### 2.8. Measurement of pH

Optimized NE's pH was calculated by using a pH meter (EUTECH Instruments, Singapore) at 25 ± 2°C in triplicate [29].

### 2.9. Transmission Electron Microscopy (TEM)

Using transmission electron microscope (TEM) (CM 200, Philips Briarcliff Manor, NY, USA) operating at 200 KV, surface morphology of NRG NE was studied. NE was adequately diluted with water (1:1000) and a drop was applied to a 300 mesh carbon coated copper grid of 400 mesh. Further, it was stained phosphotungstic acid (PTA) (2 % (w/v)) and leaving as such for 1 min to air dry. The dried grid was further examined for size, character, and form of NE [12].

### 2.10. *In Vitro* Release Study

*In vitro* release studies were executed to evaluate the release of NRG from NE formulation with that of the NRG suspension. Trials of NRG NE (2 ml; containing 40 mg/ml of NRG), along with a NRG suspension (2 ml; containing 40 mg/ml of NRG), were carried out using a dialysis membrane method. The dialysis bag (having 12 000 g/mol molecular weight) was pretreated as per the provided standard method and the apparatus was set at 100rpm maintained at 37 ± 2°C over a magnetic stirrer (Remi Instrument Ltd., Mumbai, India). 2 ml of each formulation was placed in different bags and dipped in 100ml of phosphate buffer pH 6.4 as this corresponded to the pH of nasal fluid. 1 ml of the sample was withdrawn at different time points (0, 30, 60, 90, 120, 150, 180, 210, 240, 270, 300, 360, and 420 min), and the same amount of fresh media was added to maintain sink conditions. Samples were analyzed for NRG content. The samples were examined at 322 nm by UV spectrophotometer (Shimadzu Corp, Kyoto, Japan) after appropriate dilution. All measurements were done in triplicate.

### 2.11. *Ex Vivo* Nasal Mucosa Permeation Study

*Ex vivo* permeation studies were carried out using a Franz diffusion cell (15.2 ml receiver volume). NRG NE was compared with the NRG suspension using on freshly isolated goat nasal mucosa collected from slaughter house. The goat mucosa was stored in a 10% formalin solution in a deep freezer (−80°C) till further use. Nasal membrane was carefully detached and was wiped with isopropyl alcohol making it free from adhered tissues. 0.2 mm thick tissue was taken and mounted between the donor and receiver compartment of the Franz diffusion cell. Tissue was mounted so that mucosal side should face the donor compartment while the dermal side will be faced on the receiver compartment. Using dissolution medium in the running media (phosphate buffer pH 6.4), mucosa was made stabilized. It was repeated for 10 times by changing the media after each run at 37 ± 1°C. 1 ml of the selected NRG suspension and NRG NE was placed onto each donor compartment, respectively. At different time points (0, 30, 60, 90, 120, 150, 180, 210, 240, 270, 300, 360, and 420 min) samples were withdrawn and the same amount of fresh media was added. Samples were then analyzed for content of NRG. After appropriate dilution, using UV spectrophotometer (Shimadzu Corp, Kyoto, Japan) samples were investigated at 322 nm. All measurements were done in triplicate.

### 2.12. Antioxidant Activity (DPPH Method)

This test is based on the free radical scavenging activity of the stable DPPH free radical. In DPPH assay method, ascorbic acid (AA) was considered to be a standard antioxidant. Antioxidant activity of vitamin E, NRG NE, and NRG suspension were compared with that of AA. Vitamin E, NRG suspension, and NRG NE and AA were diluted using methanol in a serialized dilution from 1–25 *μ*g/ml. 1 ml of DPPH solution (0.004% w/v) in methanol was added to 1 ml of each sample. After 30 min, using blank as methanol absorbance was measured at 515 nm [20]. Following formula was used to calculate the percentage inhibition:

(1) $$\begin{array}{c} \%\;\;Inhibition=\frac{A_{0}-A_{1}}{A_{1}}\;\;X\;\;100 \end{array}$$

In the above equation, A_0_ signifies the blank's absorbance and A_1_ signifies the drug solution's absorbance. Further a plot was constructed between mean % inhibition and log concentration. By interpolation using a graph pad (Prism 6 software, San Diego, California), 50% inhibitory dose (IC50 value) was calculated which was then compared against the standard.

### 2.13. Pharmacokinetic and Brain Targeting Study

For the pharmacokinetic studies, Wistar rats of either sex, ageing between 11 and 12 weeks and weighing between 200 and 250 gm were taken. Institutional Animal Ethical Committee (AEC), Jamia Hamdard, New Delhi, India, has approved the animal study protocol (173/Go/Re/ S/2000/CPCSEA; Approval no. 1176, 2015). Guidelines provided by the AEC were followed during the studies. Further, the animals were divided into different 3 groups, i.e., Groups A, B, and C (each group having 3 rats). Group A was administered with NRG solution via intranasal route (dose 0.72 mg/kg/day dissolved in 0.5 ml of normal saline solution); Groups B and C were administered with NRG NE via intranasal route and intravenous route (dose equivalent to 0.72 mg/kg/day), respectively. Blood samples (0.2 ml) were collected by cardiac puncture and the animals were sacrificed at 0.5, 1, 2, 4, and 8 h time intervals. The blood samples were collected in the microcentrifuge tubes containing EDTA (an anticoagulant) which were then centrifuged for 20 min at 5000 rpm. The supernatant so obtained was stored in deep freezer (−70 ± 10°C) for further HPLC estimation. After collection of the brain it was washed with normal saline to make it free from adhering tissue/fluid. Then it was placed in methanol. Concentration of the drug in brain and plasma was estimated using pharmacokinetic software (PK Functions for Microsoft Excel, Pharsight Corporation, Mountain View, CA). Various parameters like AUC, C_max_, T_max_, DTE % (drug targeting efficiency), and DTP % (direct transport percentage) were further calculated. C_max_ (maximum plasma concentration) and T_max_ (time required to reach the maximum concentration) were estimated directly from the real plasma profiles. By using linear trapezoidal method, AUC (area under curve) was computed [29, 30]. The brain targeting efficiency was estimated by the use of following equations:

(2) Drug  tageting  efficiency DTE%=AUC  brain/AUC  bloodi.n.AUC  brain/AUC  bloodi.v.X  100Drug  transport  percentage DTP%=Bi.n.−BxAUC  brain/AUC  bloodi.v.X  100

where B_x_ is the brain AUC fraction contributed by systemic circulation through the BBB following i.n. administration which is calculated as follows:

(3) $$\begin{array}{c} B_{x}=\left(\;\frac{B_{i.v.}}{P_{i.v.}}\;\right)\;\;X\;\;P_{i.n.} \end{array}$$

B_i.v._ and P_i.v._ are the AUC_0–480_ for brain and Plasma following intravenous administration, respectively.

B_i.n_. and P_i.n._ are the AUC_0–480_ for brain and plasma following intranasal administration, respectively.

The %F (percentage absolute bioavailability value) of the i.n. formulations was calculated as follows:

(4) $$\begin{array}{c} \%F=\left(\;\frac{{AUC}_{i.n.}}{{AUC}_{i.v.}}\;\right)x\;\;\frac{{Dose}_{i.v.}}{{Dose}_{i.n.}}\;\;x\;\;100 \end{array}$$

### 2.14. Biodistribution Studies

Confocal laser scanning fluorescence microscopy (CLSM) using a fluorescent dye (i.e., ROD-123) was used to study the biodistribution of NRG NE and NRG solution. This fluorescent dye is used as it has less capability to cross the BBB even when given via intravenous route. For using the dye in the formulation, firstly ROD-123 was dissolved in ethanol (20 mg/ml) which was further blended with the formulation (0.175% w/v). Wistar rats of either sex having weight between 200 and 250 g were selected which were further divided into 3 groups (each group consisting of 3 animals). Group I was given ROD-123 loaded NRG solution via intranasal route, and Groups II and III were loaded with ROD-123 NEs via intranasal and intravenous route, respectively. Using cervical dislocation method, animals were humanely sacrificed at 0.5 h after which brain was dissected. The brain was made free from the adhering tissue/fluid by washing it twice using normal saline. Using a microtome, brain was cut into 5*μ*m thickness to get it fixed in 4% (w/v) formaldehyde solution on a slide which was stored at 4°C before the commencement of CLSM studies. These slides were further examined by means of a fluorescence microscope (Olympus FluoView™ FV 1000, CA, USA). Red fluorescent dye ROD-123 leaves the red fluorescent spots which were judged as the noticeable markers of the drug entrenched in NEs [20].

### 2.15. Nasal Ciliotoxicity Studies

Freshly isolated nasal mucosa of goat was obtained from slaughter house and was kept in phosphate buffered saline (PBS pH6.4) to perform the nasal ciliotoxicity studies. Nasal mucosa was divided into 3 sections of homogeneous thickness (0.2 mm). Mucosa was then mounted on Franz diffusion cell. Out of the 3 mucosal pieces first piece of mucosa (M_1_) treated with isopropyl alcohol will serve as a positive control, second piece of mucosa (M_2_) was treated with PBS of pH6.4 (serving as negative control) and the third mucosal piece (M_3_) was treated with drug loaded optimized NE. After 2 h of treatment, samples were rinsed with PBS (pH6.4) appropriately which were further stored 10% formalin to be carried to the pathological laboratory for the slide preparation. A 5 *μ*m thick mucosal piece was cut with the help of a microtome which was then stained with hematoxylin and eosin. Further, the pieces were studied under light microscope joint with an image processor (Motic, Nagoya, Japan) at a magnification of 40x. They were studied for the toxicity to nasal mucosa and the images were documented [31, 32].

### 2.16. Pharmacodynamic Studies

#### 2.16.1. Animals and Treatments

The rats (weighing 200-300 gm) were randomly divided into 5 groups (each group having 6 animals); namely, Group A was treated with normal saline via intranasal route served as a control, Group B was a 6-OHDA induced group again treated with normal saline intranasally served as lesioned control group (PD Model), Group C was a 6-OHDA induced group treated with NRG NE intranasally, Group D was 6-OHDA induced group treated with marketed formulation (levodopa) orally, and Group E was 6-OHDA induced group treated with NRG NE intranasally along with oral levodopa. Rats were anesthetized using ketamine/xylazine cocktail (0.1mL/20g rat wt. IP). The duration of anesthesia was 20-30 minutes. To validate the adequate anesthesia depth, corneal and gross motor reflexes were monitored. The animals were held onto a stereotaxic frame (dual manipulator model 51600 Stoelting Co., IL, USA). Nonpuncture ear bars of 45 mm having a nose bar position of 2.3 mm beneath the interaural line were used. Animals were mounted on a stereotaxic stand and a slight incision was made of skull's skin of the skull for the exposure. The striatum coordinates were calculated precisely as for rat 1.0 mm of anteroposterior (AP), lateral (L) 2.5 mm to the right side, dorsoventral (DV) 4.5 mm relative to bregma, and ventral from dura with the tooth bar set at 0 mm. Subsequently, rats were lesioned into the right striatum and were injected with 10 *μ*g 6-OHDA/2*μ*L mixed with 0.1% ascorbic acid-saline. Group A was injected with 2.0 *μ*l of the vehicle only i.e., 0.1% ascorbic acid-saline. Automatic microdrilling machine was used which was attached on stereotaxic apparatus to made burr holes in skull for injecting the vehicle at a rate of 0.5 *μ*l/min manually using a Hamilton syringe. After the injection needle was held in the same place for further 5.0 min allowing the toxin to diffuse away from the lesion site. Recovery from anesthesia approximately took 4–5 h. Individual cages were used to keep the treated rats in a well aerated room at 25±2°C until they gain full consciousness. Afterwards they were housed together in groups as specified above [33].

#### 2.16.2. Narrow Beam Maze (NBM) Test

Animals from all the groups were trained on a narrow beam maze before the lesioning for their balance and motor coordination. NBM consists of a start-and-goal box having 13 cm width and 40 cm high walls and a wooden narrow beam runway of 180 cm in length and 2 cm in width. The beam was made 1 meter elevated from the ground with extra supports. The inner walls of the start-and-goal box were painted black. For 3 consecutive days, animals were tested on NBM for 10 trials per day, each trial having interval of 1 min. During the experiment food pellets were placed in the goal box for the motivation of animals. The time taken by the animals to reach the goal box from the start box was noted [34]. The same procedure was repeated on the 16^th^ day after lesioning for 1 day and time taken by the animals to complete their journey from start box to the goal box was measured. After 27 days of lesioning, animals were treated as per the schedule and the same procedure was repeated and time was noted.

#### 2.16.3. Muscular Coordination Test

To study the motor-coordination ability, the rotarod experiment was performed. Animals from all the groups were trained before the lesioning on rotarod apparatus, i.e., rotarod unit (Omni Rotor, Omnitech Electronics, Inc., Columbus, OH, USA). Rats were allowed to retain on a rotating rod having 75 mm diameter. The retained time for each animal on the rotating rod was recorded having minimum interval between each of 5 min and maximum trial length of 180s. The trials were done in triplicate. The time in tenths of a second was automatically recorded by the apparatus till the rat falls on the floor. The speed was set at 10 rotations per minute having 180 s as cut-off time. The score was presented as mean of latencies of three trials on the rotating rod [34]. Data were accessible as retention time on the rotating bar over the three test trials. After lesioning the same procedure was repeated on the 16^th^ day and after 27 days. Animals were treated as per the schedule and the same procedure was repeated and time was noted.

#### 2.16.4. Forced Swimming Test

Animals from each group were trained for swimming test by placing each rat in a bucket (46 cm diameter X 60 cm height) filled with fresh water (temperature: 28 ± 2°C) 30 cm deep. Data like the mean swimming time, climbing time (trying to climb the wall of bucket), and immobility time (staying afloat and exhibiting minimum movements) were recorded [35]. After lesioning the same procedure was repeated on the 16^th^ day and after 27 days. Animals were treated as per the schedule and the same procedure was repeated and time was noted.

#### 2.16.5. Grip Strength Test

The string test was used to measure grip strength and limb impairment. The apparatus consists of a string measuring approximately 50 cm in length which is pulled tight between two vertical supports and at a height of 40 cm from a flat surface. The rats were made to hang on the string at midway and then scored per the mentioned scale.

If the rat was unable to hang and fall off, it will be given 0; if the rat hangs onto string by two forepaws, it will be scored as 1; if the rat hangs onto string by two forepaws trying to climb on string, it will be marked as 2; if the rat hangs onto string by two forepaws along with one or both hind paws, it will be scored 3; if the rat hangs onto string by all forepaws along with tail wrapped around the string, then it will be given 4 score; and if the rat manages to escape the string, it will be scored as 5. The results were expressed as the total score [36]. The rats were trained for 3 times a day for 3 days. After lesioning the same procedure was repeated on the 16^th^ day and after 27 days. Animals were treated as per the schedule and the same procedure was repeated and time was noted.

#### 2.16.6. Akinesia Test

The delay in initiating the movement may be estimated via performing the akinesia test. This is a general abnormality in motor function in PD. The test is very simple, rapid, and easy to conduct. Also, it is helpful in giving the information related to the upper limb motor function. The test was commenced as described by Olsson and coworkers and the latency was recorded in terms of time needed (in seconds) by rat to initiate the movement. The test was concluded when latency surpasses 180 s [29]. The time taken by each animal to initiate movement for all four limbs was observed and recorded.

#### 2.16.7. Biochemical Estimation

*Tissue Preparation*. Brain tissue is used to study the enzymatic activity. 200 mg of brain from each rat was extracted and was subjected to homogenization for 3 minutes with the help of a tissue homogenizer. It gives 10 % (w/v) homogenate in 0.1 M potassium phosphate buffer (pH 7.4) containing 1 mM ethylenediaminetetraacetic acid (EDTA). Further this homogenate was centrifuged for 5 min at 800 rpm at 4°C to separate the nuclear debris. Supernatant so obtained was again subjected to centrifugation for 20 min at 4°C at 10,500 rpm so that a postmitochondrial supernatant (PMS) is obtained.

*Glutathione (GSH) Estimation*. GSH was estimated as the total nonprotein sulphydryl groups, 5,5′-dithio- (2-nitrobenzoic acid) (DTNB) is reduced by the –SH groups of GSH to form one mole of 2-nitro-5-mercaptobenzoic acid per mole of –SH. The intense yellow color of the released nitromercaptobenzoic acid anion was used to measure –SH groups at 412 nm. 2 mL of 10% of the brain homogenate was mixed with 2 mL of 0.02M (EDTA) solution which was further diluted with 1.6 mL of distilled water and 0.4 mL of 50% trichloroacetic acid (TCA) solution. The mixture was then shaken for 15 min and was subjected to centrifugation at 3000 rpm for 15 min. After the centrifugation, supernatant was separated out from which 2 mL of was mixed with 4 mL of Tris buffer (0.4 M, pH 8.9) and 0.1 mL of DTNB solution. The obtained yellow colored complex was studied at 412 nm spectrophotometrically and the content for GSH content was articulated as micromoles of GSH per milligram of protein [29].

*Thiobarbituric Acid-Reactive Substances (TBARS) Estimation*. Estimation of lipid peroxidation rate was done by mixing 2 mL of potassium chloride (0.15 M) with 2 mL of 10% brain homogenate. It was then homogenized for 3 min using a tissue homogenizer. 1 mL of the homogenate was pipette out in a test tube with further addition of 0.5 mL TCA (30%) followed by 0.5 mL (thiobarbituric acid) (TBA, 0.8%). Mixture so obtained was kept in a water bath for 30 min at 80°C followed by keeping it in ice cold water for the next 30 min. This mixture was again subjected to centrifuge for 15 min at 3,000 rpm for 15 min. Supernatant so obtained was collected in a test tube for which absorbance was read at 540 nm. In the same way, 1 ml of brain homogenate was centrifuged for 10 min at 5000 rpm from which 0.5 mL of the supernatant was separated out which was further mixed with 2.5 mL alkaline copper sulfate for the protein estimation. The mixture so prepared was made to rest at room temperature for 10 min followed by addition of 0.25 mL of folin reagent which was estimated at 750 nm spectrophotometrically after 30 minutes [29].

*Superoxide Dismutase (SOD) Estimation*. A quantity of 2 mL of 10% of the brain homogenate was mixed with 2 ml of the potassium phosphate buffer pH 7.4. The prepared mixture was centrifuged for 10 min to maintain the temperature at 4°C at 1,000 rpm. 3 mL of Tris HCL buffer was added to 100 *μ*L of supernatant followed by the addition of 25 *μ*L pyrogallol. The solution obtained was estimated spectrophotometrically at 420 nm [29].

### 2.17. Statistical Analysis

All samples used in the study were freshly prepared and the experiments were carried out in triplicate. Statistical analysis of data was carried out using computer software graph pad Prism 7.0 (Graph Pad Software San Diego, CA). All experimental data were reported as mean ± standard deviation (SD). One-way ANOVA followed by Dunnett's t-test was used for analysis of the result. The statistical implication level was set at p < 0.05.

## 3. Results and Discussion

### 3.1. Analytical Methodology

The retention time of NRG was found at 8.34 min. By using the standard deviation method, limit of detection (LOD) and limit of quantification (LOQ) were calculated and found to be 23.34 ng/ml and 67.88 ng/ml, respectively.

### 3.2. Excipients Selection

**Figure 1:**
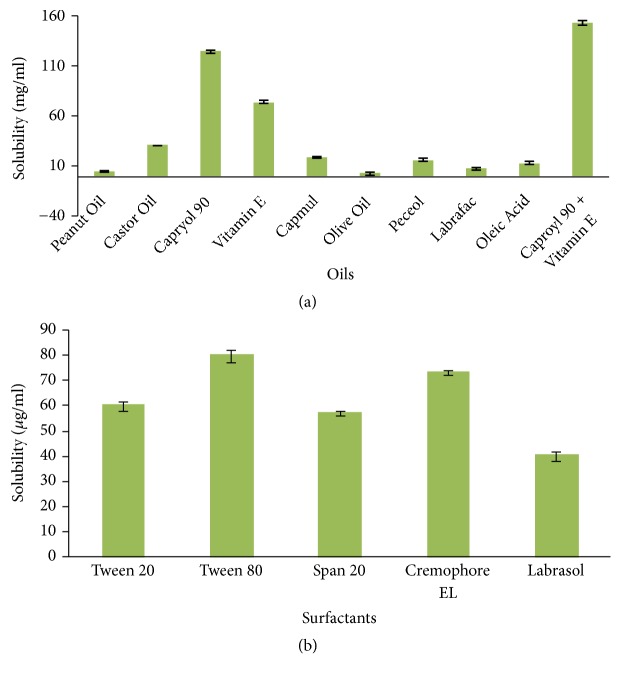
Shows solubility of NRG in different (a) oils (n=3, mean ± SD) and (b) surfactants (n=3, mean ± SD).

Oil being the solubilizing agent for lipophilic drugs is the chief component in formulating the NE. The drug should be freely soluble in the oil to avoid any forced solubility which may result in drug's precipitation above a time period. Solubility studies demonstrated that NRG is highly soluble in the combination of Capryol 90: vitamin E (1:1), i.e., 152.98 ± 2.42 mg/ml (Figure 1). Vitamin E was selected as it along with serving as an oily phase may also boost the antioxidant activity. Surfactant selection is also a critical parameter such that decreasing the interfacial tension the concentration should not cause any toxicity [37]. Based on this, nonionic surfactants are generally considered to be less toxic than the ionic surfactants [38]. On the basis of solubility and miscibility studies, Tween 80 was selected as the surfactant to be used. In addition to belonging to a nonionic class of surfactant Tween 80 is also less distressed by ionic strength and pH (Figure 1). Surfactant alone is unable to achieve the transient negative interfacial tension and fluid interfacial film, because of which a cosurfactant needs to be added as it reduces the bending stress making the interfacial film flexible. Based on the miscibility studies, Transcutol-HP was selected as the cosurfactant (Table 1). All the chemicals used in the study were nonirritant and nonsensitizing to the skin. Also, the chemicals were pharmaceutically acceptable and were generally regarded as safe (GRAS category) for use.

### 3.3. Pseudoternary Phase Diagram Construction

**Figure 2:**
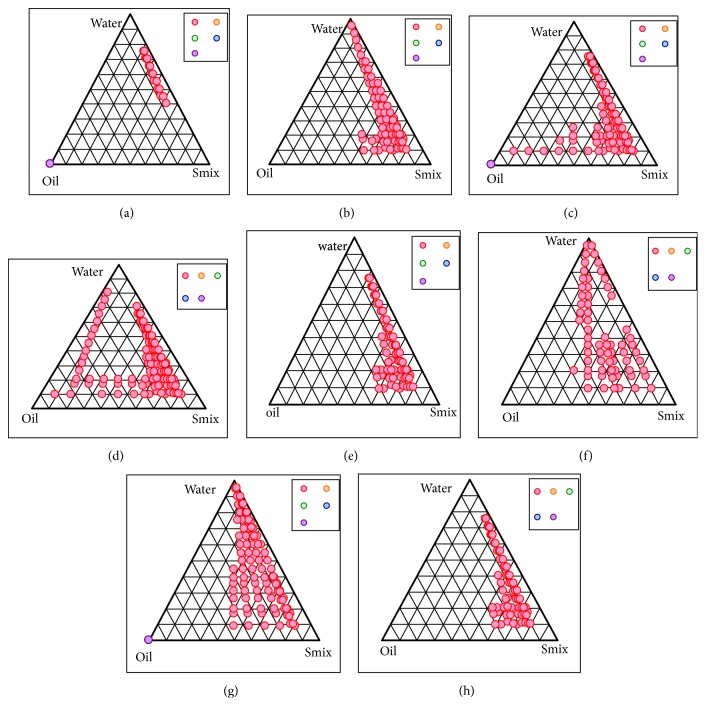
Pseudoternary phase diagrams system containing the following components: Capmul MCM as oil, Tween 80 as surfactant, and Transcutol-HP as cosurfactant. Dotted area shows O/W NE region in different ratio of surfactant to cosurfactant. (a) S_mix_ (1:0); (b) S_mix_ (1:1); (c) S_mix_ (1:2); (d) S_mix_ (1:3); (e) S_mix_ (2:1); (f) S_mix_ (3:1); (g) S_mix_ (4:1); (h) S_mix_ (5:1).

Using aqueous phase titrations, pseudoternary phase diagrams were constructed to determine the surfactant to cosurfactant ratio competent of constructing the largest isotropic NE region. Pseudoternary phase diagram were constructed using oil phase of capryol 90: vitamin E (1:1), Tween 80 as the surfactant along with cosurfactant as Transcutol-HP and aqueous phase as distilled water. The area of NE was found in order of S_mix_ 4:1>3:1>2:1>1:1>1:0. It was observed that the small or large area of NE is directly dependent on the S_mix_ ratio and its ability to solubilize the oily phase along with decreasing free energy of the system. From the figure it was observed that less NE area is shown in the S_mix_ ratio 1:1 followed by 1:2 (Figure 2). As compared to the S_mix_ ratios 2:1 and 3:1, 4:1 showed the maximum NE region based on its ability to solubilize the oil phase while decreasing the system's free energy indicating the increasing emulsification with increasing concentration of surfactant. On further increasing the S_mix_ ratio to 5:1 it was observed that area was slightly decreased signifying that the further addition of the surfactant does not contribute to the emulsification process. Hence, 4:1 of the S_mix_ was selected as the NE region.

### 3.4. Physical Stability Testing of NEs

NEs are the thermo-kinetically stable systems which are prepared at a particular concentration of S_mix_, oil separation and water. The formulation so prepared should be devoid of creaming, cracking and phase. As mentioned earlier the chosen formulations were passed through different stress conditions, namely, heating-cooling cycle, freeze thaw cycle, and centrifugation (Table 2). Results demonstrated that the formulations prepared from the S_mix_ ratio of 1:2 and 1:3 was not able to pass the stress test performed to assess the physical stability owing to the formulation's insufficient emulsification. A number of formulations turn turbid, the possible reason of which might be Ostwald ripening.

### 3.5. Preparation of Drug Loaded NE

For the preparation of the drug loaded formulation, NRG was dissolved in the oil phase. This was followed by addition of the required quantity of S_mix_ and drop wise addition of distilled water until a clear and transparent liquid was attained (Table 3).

### 3.6. Globule Size and Surface Charge Analysis

**Figure 3:**
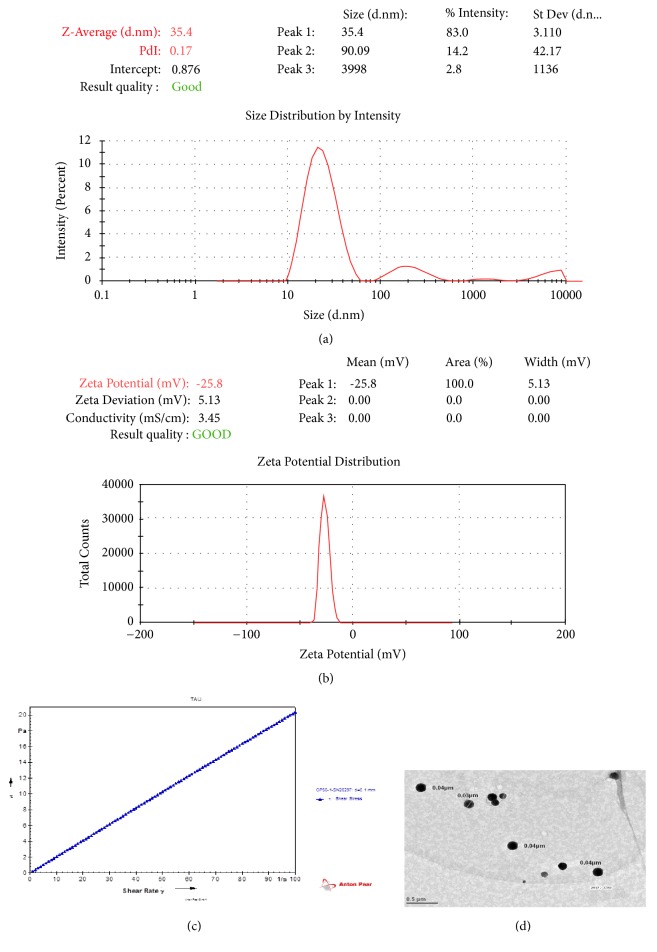
(a) shows the particle size of the optimized formulation; (b) shows the Zeta potential of the optimized formulation; (c) shows the Rheogram of optimized NE; (d) shows the TEM image of the optimized formulation.

Optimized NE was transparent and monophasic having the mean globule size of 38.70 ± 3.11 nm (n=3) having a uniform PDI of 0.14 ± 0.0024 (Figure 3(a)). It may be due to the larger emulsification area provided by the S_mix_. The higher the concentration of the surfactant stronger is the stabilization because of the closely packed surfactant film present at the oil-water interface. This suggests that the surfactant has more important role to play in nanoemulsification of the oily mixture than the cosurfactant. Also, lesser the PDI better is the size distribution of the globules along with the stability of the formulation [39]. Optimized formulation had a zeta potential of -27.4 ± 0.14 mV indicating good dispersion stability and also ensuring the creation of a high-energy barrier against coalescence of the dispersed globules (Figure 3(b)).

### 3.7. Refractive Index and Viscosity Studies

*Isotropic nature of the formulation is demonstrated through the RI value of it. RI also explains the chemical interaction between the drug and the excipients*. There were no significant differences between RI of drug loaded NE and the placebo formulation indicating the chemical stability and isotropic nature of the NE. RI of the optimized formulations was found to be 1.43 ± 0.01. While optimizing the intranasal formulations, viscosity plays a vital role as it is directly related to the residence time in the nasal mucosa. Hence in order to have a hassle free administration and better penetration and to surmount mucociliary clearance, optimum viscosity of the formulation is desired. Using an Abbe's-type refractometer (Guru Nanak Instruments, New Delhi, India), viscosity of the NE was found to be 19.67 ± 0.25 Pa s. Newtonian type of flow characteristic of NE was inferred based on the linear correlation between shear rate and shear stress over a wide range of 0-100 s^−1^and 0-20 Pa (Figure 3(c)).

### 3.8. Percent Transmittance

Transmittance of the developed formulation was found to be 98.12 ± 0.07 % indicating the stability of the NE. NEs transmittance was almost equal to the aqueous phase transmittance that confirms that oil droplet was uniformly distributed with continuous phase. The formulation is transparent because the maximum size of the globules of dispersed phase was found no larger than the 1/4th of visible light's wavelength [40].

### 3.9. Transmission Electron Microscopy

Morphology of the prepared NE was analyzed via TEM studies. It was found that the optimized formulation had spherical globules having size of 35.43 ± 4.10 nm without any sign of aggregation (Figure 3(d)). Globule size measured via TEM analysis was in compliance with the one measured through zetasizer.

### 3.10. Measurement of pH

pH of the optimized NE was found to be 6.2 ± 0.7. The pH of the NE was in adequate range for nasal drug delivery system signifying its nonirritant nature.

### 3.11. DSC Studies

**Figure 4:**
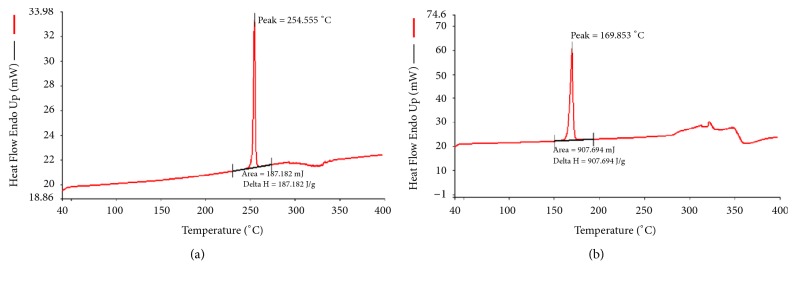
The DSC thermograms: (a) pure NRG; (b) optimized NE.

DSC thermograms were obtained for pure NRG, placebo NE, and drug loaded NE (Figure 4). Pure NRG showed a sharp melting endotherm peak at 254.56°C. Thermograms of the placebo and NRG loaded NE showed a broad asymmetric endothermic peak at approximately 168°C, which may be due to evaporation of water [41]. DSC of the NRG loaded NE indicates that the drug was molecularly dispersed in the oily phase of formulation and hence the peak of the drug was not observed.

### 3.12. *In Vitro* Release Study

**Figure 5:**
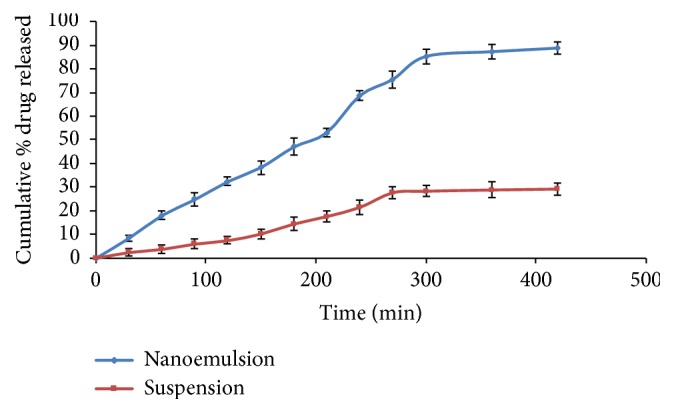
*In vitro* comparative release profile of NRG suspension and NRG loaded NE.

*In vitro* release studies were carried out to compare the release of NRG from NRG NE and NRG suspension. The studies revealed that the drug release from NRG NE was significantly higher in comparison to NRG suspension (p < 0.05). The cumulative percentage release of NRG from NE was found to be faster i.e., 88.875 ± 2.587 % over a period of 8 h in comparison to the release from NRG suspension having only 29.118 ± 2.787 % drug release after 8 h. Also, drug precipitation was observed in the dialysis bag of NRG suspension (Figure 5).

The data so obtained were further fitted to various release kinetic models, namely, zero order, first order, Higuchi, and Hixson-Crowell model to identify the drug release mechanism from NE. The results demonstrated that drug release from NE follows zero order model owing to the highest value of coefficient of correlation (R^2^), i.e., 0.937, which is nearer to unity (Table 4).

### 3.13. *Ex Vivo* Nasal Mucosa Permeation Study

**Figure 6:**
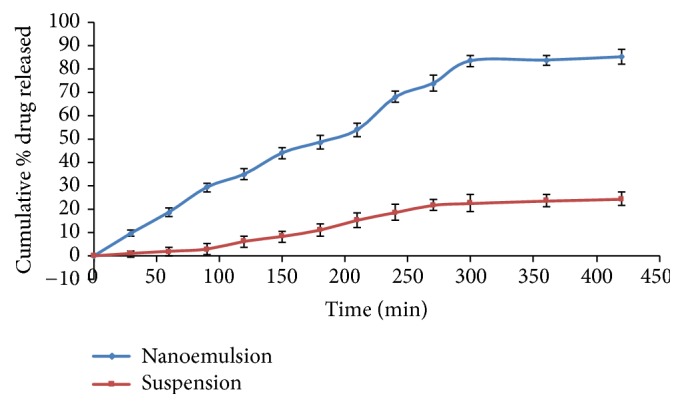
*Ex vivo* comparative release profile of NRG NE and suspension.

Nasal mucosa permeation studies were conducted to evaluate the permeation from the NRG NE and NRG suspension. Goat nasal mucosa was used that acted as a diffusional barrier mounted on Franz diffusion cell. NRG loaded NE showed maximum permeation of 85.45 ± 3.404 % at 8 h as compared to the NRG suspension which showed 24.644 ± 3.107 % of permeation (Figure 6). Drug was rapidly removed from nasal mucosa because of the mucociliary clearance ultimately causing the high permeability across the nasal mucosa which is advantageous* in vivo*. After 8 h of study, steady-state flux and permeability coefficient of NRG suspension and NE through nasal mucosa were calculated to be 20.88 *μ*gcm^−2^h^−1^, 9.07×10^−1^cm^−2^h^−1^ and 64.78 *μ*gcm^−2^h^−1^, 28.16×10^−1^cm^−2^h^−1^, respectively. The flux and permeability coefficient of NE were almost three times in comparison to the NRG suspension. The drug release pattern from the* ex vivo* permeation studies was in analogy with that of the* in vitro* studies. Thus, from the above study, it may be accomplished that the smaller the globule size of the formulation is, the higher its drug permeation through the nasal mucosa will be.

### 3.14. Antioxidant Assay by the DPPH Method

**Figure 7:**
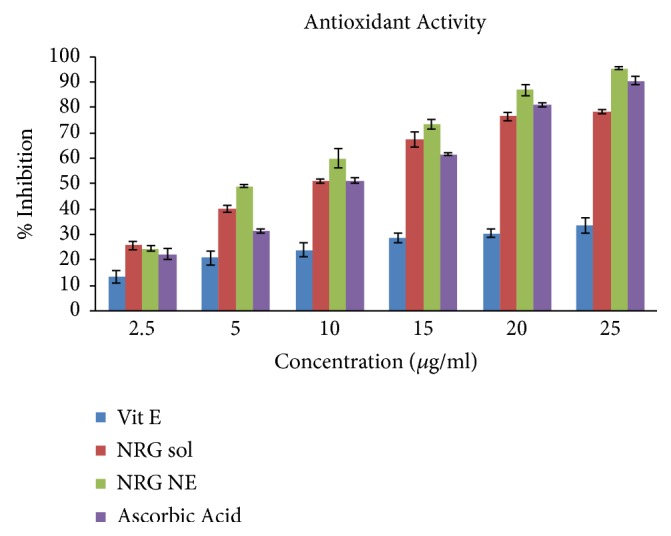
Comparison of antioxidant activity of vitamin E, ascorbic acid, NRG NE, and NRG solution by DPPH assay.

NRG has hydrogen donating capability due to which it shows the antioxidant activity on DPPH radical. NRG efficacy was estimated via determining the decline in absorbance at 515 nm. It may also be noticed visually based on the color change from purple to yellow. NRG has the ability to scavenge the free radical which decreases the concentration of DPPH. Figure shows that optimized NE exhibits the maximum percentage inhibition (95.28 ± 0.64 %) followed by the ascorbic acid (90.44 ± 1.56 %) and the NRG solution (78.32 ± 0.81 %) (Figure 7). It may also be inferred that the significant antioxidant activity (p < 0.05) of the optimized NE may be due to the synergistic effect of the combination of NRG and vitamin E.

### 3.15. Pharmacokinetic and Brain Targeting Studies

Pharmacokinetic parameters were estimated through the i.n. and i.v. administration of the optimized formulation along with the NRG solution via i.n. route. NRG concentration in the brain via optimized NE was found to be extensively elevated (p < 0.05) at all given time points when compared to the NRG NE and solution via i.v. and i.n. route, respectively. After the 30 mins of administration of optimized NE i.n. and i.v and NRG solution i.n., the brain concentration was calculated to be 394.33 ± 80.83 ng/ml, 179.94 ± 66.50 ng/ml and 298.40 ± 71.50 ng/ml respectively (Table 5). The results thus clearly indicated that the NRG concentration is significantly higher in optimized formulation administered via i.n. (p<0.05) than that of administration of optimized formulation via i.v. and NRG solution via i.v. route. The brain/blood ratios of NRG for the optimized NE i.n. and i.v and NRG solution i.n. was found to be 3.69 ± 0.25, 0.07 ± 0.25 and 2.66 ± 0.68, respectively after 30 mins indicating the superiority of optimized formulation for direct nose-to-brain delivery of NRG circumventing the BBB. DTE% and DTP% of the optimized NE administered via i.n. were found to be 822.71 ± 9.14 and 72.14 ± 5.87 %, respectively. It was considerably higher (p<0.05) in comparison to the DTE% and DTP% of NRG solution (666.51 ± 8.95 % and 68.41 ± 7.42 %, respectively) given via i.n. route. Also, the AUC_0–480_ of brain concentration of NRG loaded NEs (i.n.) (5345.13 ± 7.5 ng/ml*∗*h) was considerably higher (p<0.05) than the AUC_0–480_ of NRG solution (i.n.) (3352.86 ± 8.9 ng/ml*∗*h). Absolute bioavailability of NRG solution (i.n.) in blood was calculated as 25.48 ± 4.71 and in brain it was calculated to be 209.63 ± 8.24%, respectively; however in NRG loaded NEs (i.n.) it was calculated to be 50.14 ± 5.37 and 334.20 ± 8.91 in blood and brain, respectively.

### 3.16. Biodistribution Studies

**Figure 8:**
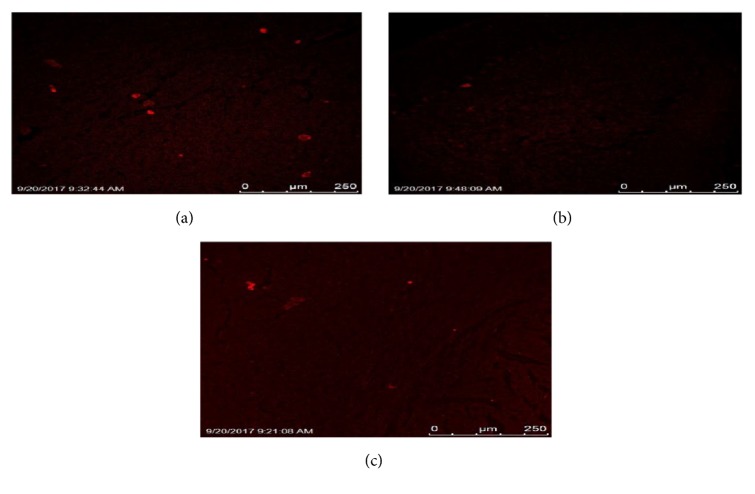
Qualitative biodistribution studies: (a) shows the ROD-123 loaded NRG NE i.n. treated brain showing the maximum intensity of dye; (b) shows the ROD-123 loaded NRG solution i.n. treated brain showing less intensity of dye as compared to the i.n. treated brain; (c) shows the ROD-123 loaded NRG NE i.v. treated brain showing the least intensity of dye.

NRG loaded NEs following i.n. and i.v. administration along with NRG solution via i.v. route were administered on Wistar rats to study the biodistribution performed by envisaging the inherent fluorescence of ROD-123 using CLSM. Confocal images of brain obtained showed that the fluorescent dye ROD-123 reached the brain in maximum intensity from NRG loaded NEs following i.n. administration followed by ROD-123 loaded NRG solution via i.n. route. ROD-123 loaded NRG NE via i.v. route showed the least intensity as shown in Figure 8.

### 3.17. Nasal Ciliotoxicity Studies

**Figure 9:**
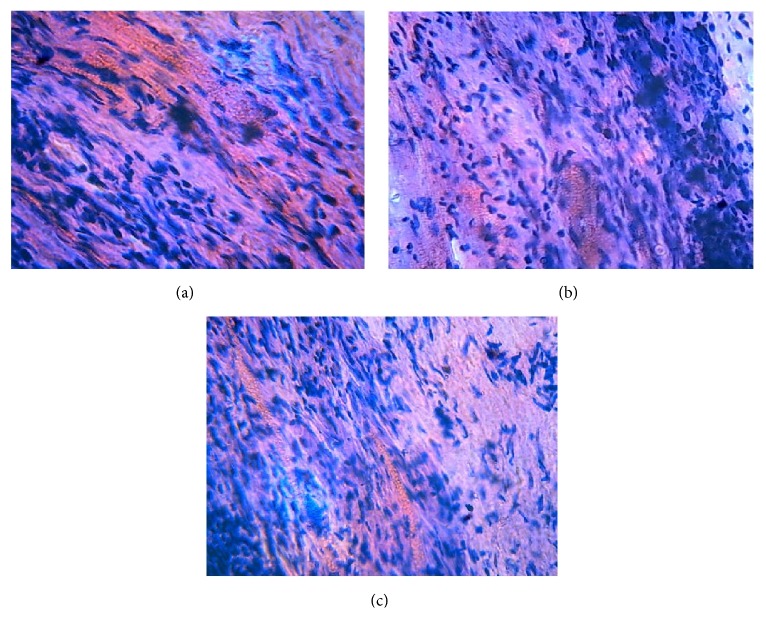
The microscopic images demonstrating the histopathological condition of nasal mucosa: (a) positive control (IPA) treated mucosa shows the damaged nasal mucosa (depicted as pink) treated with isopropyl alcohol, (b) negative control (PBS pH6.4) treated mucosa shows the intact nasal mucosa (less spots of pink) treated with isopropyl alcohol, and (c) NRG NEs treated mucosa also shows an intact epithelial layer with no traces of toxicity (less spots of pink).

Potential toxic effects of excipients used in the formulation were evaluated using nasal mucosa of goat. Nasal ciliotoxicity studies depicted that the mucosa treated with negative control (PBS pH 6.4) confirms no nasociliary damage and have an intact epithelial layer whereas mucosa which is treated with positive control (isopropyl alcohol) shows extensive damage (Figure 9). On the other hand, optimized formulation also showed an intact epithelial layer with no traces of toxicity.

### 3.18. Behavioral Activities

#### 3.18.1. Narrow Beam Maze Test

**Figure 10:**
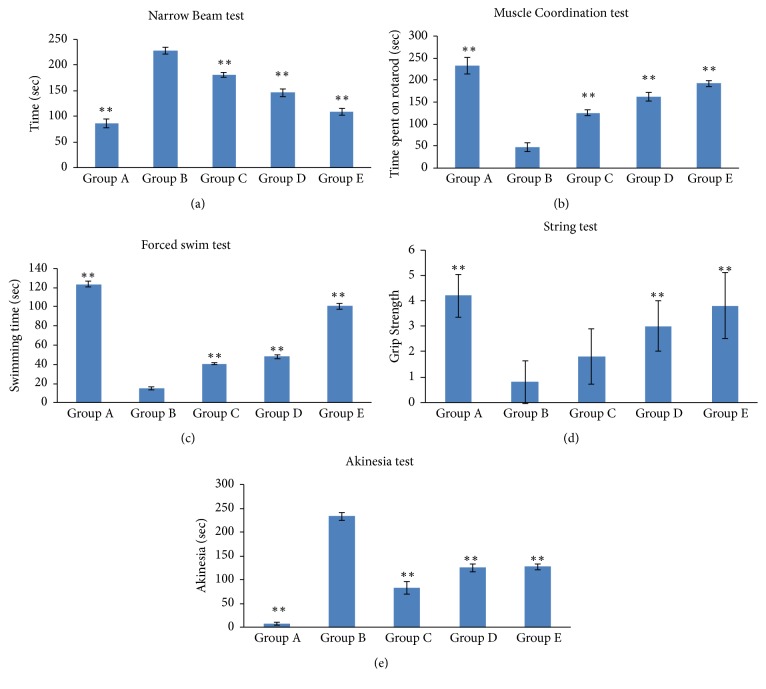
Different test performed on the different groups was depicted by histograms: (a) narrow beam test; (b) muscle coordination test; (c) forced swim test; (d) grip strength test; (e) akinesia test (**∗** comparison between Group B and other groups). Group A: treated with normal saline via intranasal route; Group B: 6-OHDA induced group treated with normal saline intranasally (PD Model); Group C: 6-OHDA induced group treated with NRG NE intranasally; Group D: 6-OHDA treated lesion group treated with marketed formulation (levodopa) orally; Group E: 6-OHDA treated lesion group treated with NRG NE intranasally along with oral levodopa.

Narrow beam test was conducted to evaluate the coordination and balance in rats (Figure 10(a)). There was significant increase in the time taken to cross the 180 cm wooden beam path (P < 0.01) in 6- OHDA treated group along with saline (228.16 ± 6.61 s) in comparison to the group which was only injected with saline (86.33 ± 7.94 s). The 6-OHDA lesion group treated with oral levodopa and NRG NE in combination showed (P < 0.01) marked improvement (108.33 ± 6.53 s).

#### 3.18.2. Muscular Coordination Test

Rotarod test was performed to estimate the muscle coordination in the rats. Group injected with 6-OHDA showed a significant diminution in muscle coordination when compared to the group which was only treated with normal saline (p < 0.001). The maximum muscular coordination was found in the group that were injected with normal saline as they spent more time (232.5 ± 18.46 s) on the rotarod (Figure 10(b)). Muscular coordination was improved in 6-OHDA challenged group which was treated with levodopa and NRG NE simultaneously (spent 161.33 ± 10.21 s on rotarod).

#### 3.18.3. Forced Swimming Test

A forced swimming test was conducted which showed that 6-OHDA induced rats treated with optimized formulation swam for 125.33 ± 5.88 s (Figure 10(c)). The saline treated group showed the swim behavior for 123.34 ± 3.11 s whereas the 6-OHDA induced rats attained very insignificant swim behavior of 15.09 ± 1.84 s (p < 0.05). No significant swim disability was observed in the 6-OHDA induced group treated with both NRG NE and levodopa when compared to the saline treated group.

#### 3.18.4. Grip Strength Test

String test was conducted to measure the grip strength of the rats (Figure 10(d)). The study showed that the group induced with 6-OHDA demonstrated a very insignificant grip strength of 0.8 ± 0.83 (p > 0.05). The group treated with saline only exhibited the grip strength of 4.2 ± 0.83 which is almost statistically nonsignificant (p > 0.05) to the group which was treated with both levodopa and NEG NE, i.e., 3.8 ± 1.30.

#### 3.18.5. Akinesia Test

The group induced with 6-OHDA treated with saline showed the inability to move due to akinesia for 236.74 ± 3.47 s (Figure 10(e)). Rats induced with 6-OHDA only and rats induced with 6-OHDA which were treated with NRG NE i.n. and levodopa oral showed akinesia for 236.74 ± 3.47 s respectively, which were found to be statistically significant (p < 0.001). However, on administration of both oral levodopa and i.n. NRG NE akinesia time was further reduced to 77.23 ± 7.95 s (p < 0.001).

### 3.19. Biochemical Estimation

**Figure 11:**
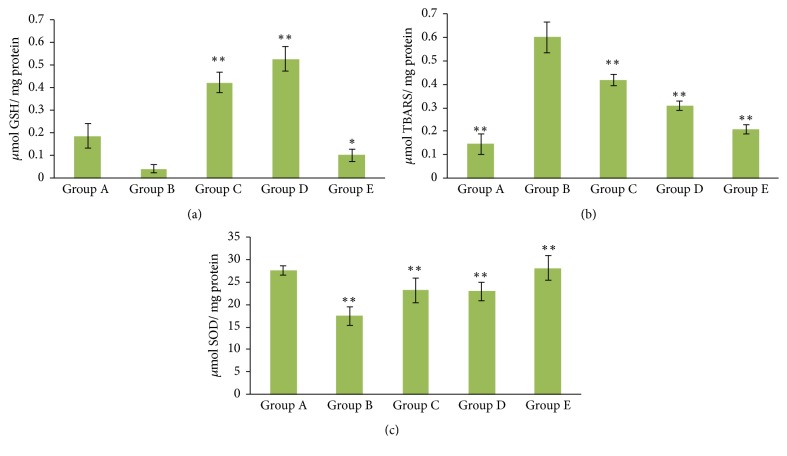
The effects of different NRG formulations with different routes on (a) GSH; (b) TBARS; and (c) SOD level in 6-OHDA induced Wistar rats (*∗* comparison between Group B and other groups).

Levels of GSH, TBARS, and SOD were compared in different formulations of NRG administered via different routes with that of the saline (Figure 11(a)). 6-OHDA induced rats showed significantly lower levels (p < 0.01) of GSH concentration (0.041 ± 0.004 *μ*Mmg^−1^) when compared to the group treated with saline (0.187 ± 0.05 *μ*Mmg^−1^). The NRG NE treated group showed slight increase in the GSH concentration (0.42 ± 0.04 *μ*Mmg^−1^); however when the rats were simultaneously treated with levodopa and NRG NE together it showed a synergistic effect with a significantly higher GSH level (0.10 ± 0.002 *μ*Mmg^−1^) (p < 0.01).

Brain tissue malonaldehyde (MDA) content was evaluated for determining the TBARS level in the brain tissue (Figure 11(b)). The MDA content in the group treated with saline only was estimated to be 0.14 ± 0.04 nM mg^−1^ protein. 6-OHDA treated group shows a considerable increase (0.60 ± 0.06 nM mg^−1^ protein) in the MDA content in comparison to the rats who were treated with saline only (p < 0.01). Rats treated with NRG NE via i.n. route showed small decrease in the MDA content (0.41 ± 0.02 nM mg^−1^ protein). Group treated with both levodopa and NRG NE showed significantly lesser MDA content (0.20 ± 0.02 nMmg−1 protein) compared to the group treated with 6-OHDA (p < 0.01).

For saline treated rats, SOD level was calculated to be 27.60 ± 1.06 *μ*Mmg^−1^ protein (Figure 11(c)). 6-OHDA induced group showed a significant dip (p < 0.01) in SOD levels (17.49 ± 2.10 *μ*Mmg^−1^ protein). 6-OHDA induced rats treated with NRG NE i.n. showed a considerable enhancement in the levels of SOD (p < 0.01) when compared to the group which were only administered with 6-OHDA. On the other hand, the group treated with both NRG NE i.n. and oral levodopa showed a significant increase (p < 0.01) in their SOD level of 28.16 ± 2.73 *μ*Mmg^−1^ protein.

## 4. Discussion

The present work was aimed to develop physically stable vitamin E loaded NRG NE to enhance its delivery to the brain via intranasal route and thus avoiding first pass metabolism and its nontargeted site circulation. Excipients were selected on the basis of solubility and miscibility studies for formulating NE. NRG was found to have the maximum solubility in a combination of Capryol 90: vitamin E (1:1) oil (i.e., 152.98 ± 2.42 mg/ml); vitamin E will have an added advantage of providing the synergistic antioxidant effect. Among the surfactants, NRG shows the maximum solubility in Tween 80 (80.52 ± 1.22 mg/ml). On the basis of miscibility studies Transcutol-HP was selected as cosurfactant as it showed miscibility with both the selected oils and surfactant. From the figures of pseudoternary phase diagrams, it was observed that area of NE is directly dependent on the S_mix_ ratio; hence 4:1 showed greatest NE region in comparison to the other ratios. On further increasing the S_mix_ ratio to 5:1 the area was somewhat decreased which indicates that further addition of surfactant will not facilitate the emulsification process. Hence, it may concluded that the NE region principally depends on the ability of a particular S_mix_ to meet the prerequisite for formulating NE, i.e., to get the oily phase solubilized and decrease the free energy of the system to extremely low level. Globule size is an important parameter to be studied for the efficient brain targeting of drugs. For an effective brain targeting size of the formulation should be less than 200 nm [42]. The mean globule size of optimized formulation was found out to be 38.70 ± 3.11 nm. From the studies it was concluded that increase in the S_mix_ ratio up to a certain level will result in the small globule size of the NE; however after which the globule size will increase with increasing S_mix_ [28]. This may be due to several factors like the increasing S_mix_ concentration up to a certain level or may be because of the decline in interfacial tension [31]. In our experiment, it was found that when S_mix_ was increased from 25*μ*l to 30*μ*l the globule size was decrease owing to the lately created droplets which have the sufficient surfactant molecules for stabilization. Alternatively, increasing the S_mix_ ratio to 35*μ*l will increase the globule size because of the presence of excessive surfactant that lowers the diffusion rate of surfactant molecules which ultimately results in the coalescence of emulsion droplets [43]. Due to excessive S_mix_ gel like clumps are formed leading to the cubic phase formation which are not easily dissolvable and also cannot exist simultaneously with the NE. Droplets of fine emulsion did not occur spontaneously due to this high viscosity [44]. In addition there is a chance of cosurfactant dominating over surfactant over a particular level of S_mix_. Similar reports have been earlier published demonstrating that upon adding surfactant to the NE system the interfacial film may condense and become stabilized, while the cosurfactant will cause the film to get expand [28].

Size distribution of NE is indicated by PDI. The PDI value of NE was found to be 0.14 ± 0.0024 respectively. Studies demonstrated that while increasing the S_mix_ concentration up to a certain level will result in the decreased PDI of NE, however on further increase the S_mix_ concentration will results in increased PDI. Additionally it may be explained that the concentration of emulsifier is inversely proportional to the flow resistance that ultimately results in the NE of higher viscosity. As a result, higher coalescence rate resulting in the larger droplet sizes [39].

Physical stability of the NE was affected by zeta potential. It was found to be -27.4 ± 0.14 mV for the optimized formulation. The negative sign is an indication of strong repulsion which ultimately prevents the particle aggregation and enhances the physical stability of NE formulation [35]. In the present study it was found that concentration of oil is directly proportional to the negative value of zeta potential, i.e., higher the oil concentration more negative will be the value of zeta potential. This may be due to the steric repulsion present at the low concentrations which gets stabilized by the addition of surfactants. It may also be concluded that as the concentration of oil was increased, amount of emulsifier or surfactant becomes insufficient (as it is responsible for the coating of droplet surface), an increase in electrostatic repulsion between oil droplets was observed which ultimately leads to the increase in the negative value of zeta potential [35].

TEM study was used to determine the morphology of the optimized NE. It showed that most of the globules were spherical in shape within the nanometer range (35.43 ± 4.10 nm). TEM images further strengthen the results as the globule's size was in accordance with the size measured by zeta sizer [3, 7]. DSC studies were performed to study the dispersion of the drug. Thermogram of the optimized formulation so obtained showed an asymmetric broad endothermic peak at 168°C. The peak may be due to evaporation of water and is indicative of the molecular dispersion of the drug in the oil phase [41].

*In vitro* release studies over a period of 8 h confirmed an initial burst release (Figure 5) and sustained releases go after. Presence of nanodroplets on the surface of NEs caused the formulation to release in bulk initially. However, the latter sustained release was because of the drug's release at oil-water interface from the oily core which was obstructed by the dialysis bag and aqueous medium and [26, 31]. The results of* in vitro *kinetic release demonstrated that drug release from NE follows zero order model owing to the highest value of coefficient of correlation (R^2^), i.e., 0.937, which is nearer to unity.

Goat nasal mucosa was used to conduct the e*x vivo* permeation studies which demonstrated that NE showed higher drug permeation through nasal mucosa in comparison to the drug solution. This higher drug permeation of NE is due to its nanodroplet size which increases the solubilization of the drug. Also, existence of a penetration enhancer i.e., Tween 80 on to the surface of NE also enhances the drug permeability via nasal mucosa [28].

The antioxidant activity of the optimized formulation and NRG solution was compared with that of the ascorbic acid (standard antioxidant) using the DPPH assay method. Optimized NRG NE showed the maximum percentage inhibition owing to the ability of NRG to donate hydrogen which showed the antioxidant effect.

*In vivo *studies verified that the concentration of NRG in brain when administered via i.n. as NRG NE was significantly higher (p<0.05) as compared to the i.n. administration of NRG solution and NRG NE administered via i.v. route. This may be due to the direct transportation of drug via olfactory route thus bypassing BBB with the usage of Tween 80 and Transcutol-HP which ultimately enhances the penetration of drug via nasal mucosa [28]. DTE% and DTP% of NRG NE was considerably higher (p<0.05) in comparison to the NRG solution suggestive of better brain targeting efficiency of NRG NE due to the considerable direct nose-to-brain delivery, nanodroplets size and lipophilicity of NE [43, 44]. There was a significant enhancement (p<0.05) in absolute BA of NRG NE via i.n. in comparison to the NRG NE via i.v. and NRG solution via i.n. owing to the small globule size, elevated drug release and increased residence time of NE in the nasal cavity. NE transportation via transcellular pathway through olfactory neurons to the brain through various endocytic pathways of sustentacular or neuronal cells in the olfactory membrane was facilitated by the small globule size of NE. Additionally, the usage of Tween-80 and Transcutol- HP in the NE considerably enhanced the nasal absorption of the NRG as a result of p-glycoprotein (P-gp) inhibition thus improved the BA of NRG [43, 44]. Hence, the present study showed that NRG could be transported directly to the brain via i.n. delivery of NRG NE, accordingly enhancing drug concentration in the brain.

CLSM study showed that the ROD-123-NRG-NE (i.n.) concentration was higher in brain as compared to the ROD-123-NRG-NE (i.v.) and ROD-123-NRG-Solution (i.n.) because of the intranasal delivery and thus circumventing the BBB. It might be due to the drug's increased permeability as lipidic nanocarrier and inhibition of P-gp efflux (by formulation excipients) which expels out ROD-123 solution [20, 30].

Nasal ciliotoxicity studies using goat nasal mucosa inveterate the presence of an intact epithelial layer, i.e., when goat nasal mucosa treated with both negative control and NRG NE it does not shows any nasociliary damage [44] (Figure 9). It is also an indicative of the excipients used in formulating NE was nonirritating, safe and nontoxic.

PD symptoms were induced using 6-OHDA in rats which were then reversed by administering NRG formulations by different routes. In the present study, there was appreciable rapid improvement in behavioral activity due to restoration of dopamine following treatment with NRG NE (i.n.). This is likely due to the antioxidant effect of NRG which ultimately leads to the reversal of PD symptoms. NE showed improved NRG brain delivery in comparison to the solution, which may be attributed to the fact that there was more drug permeation through the nasal mucosa. Small globule size of the NE offers more surface area for the interaction between the drug and the cells, hence more drug uptake through the nasal mucosa. Studies showed that ROS are involved in the pathogenesis of PD. Antioxidants help in eradicating the generated ROS. 6-OHDA induces formation of ROS in brain of rats after injection. Optimized NE formulation showed better behavioral activity via i.n. route owing to the synergistic effect of NRG and Vitamin E. Results of the present study demonstrated that NRG solution (i.v.) have no significant effect on reversal of 6-OHDA induced PD as it did not find an easy passage through BBB, on the other hand NRG solution when administered via intranasal route demonstrated effective antagonization of 6-OHDA induced akinesia, hypolocomotion and related symptoms. These interpretations confirmed the effectiveness of NRG given via the olfactory route as lower doses of the drug is required for the pharmacological actions [15, 20]. The study showed that the treatment of 6-OHDA induced rats with NE along with the standard therapy (levodopa) was successful in reversing the effects induced with 6-OHDA including muscle coordination, grip strength, enhancing swimming activity. Hence, intranasal administration of NRG NE avoids first pass metabolism thereby protecting it from the nasal metabolizing enzymes and enhancing the uptake of NRG to the brain, avoiding systemic circulation and hence enhancing the amount of NRG in the brain and improving brain bioavailability.

## 5. Conclusion

In the present study, physically stable vitamin E loaded NRG NE was formulated. Experimental data showed that there was maximum* in vitro* along with significant high* ex vivo* trans-nasal mucosal flux. Also, concentration of the drug in the brain was found to be higher proving the targeting efficiency of NE through i.n. route. The study showed that the treatment of 6-OHDA induced rats with NE along with the standard therapy (levodopa) was successful in reversing the effects induced with 6-OHDA including muscle coordination, grip strength, and enhancing swimming activity. Hence, intranasal administration of NRG NE avoids first pass metabolism, thereby protecting it from the nasal metabolizing enzymes and enhancing the uptake of NRG to the brain, avoiding systemic circulation, and hence enhancing the amount of NRG in the brain and improving brain bioavailability.

## Figures and Tables

**Table 1:** Miscibility of NRG in oil and surfactants with co-surfactants.

S. No.	Co-surfactant	Miscibility with oils	Miscibility with Surfactants
1	Transcutol HP	Clear	Clear

2	Propylene Glycol	Turbid	Turbid

3	Captex 200	Clear	Turbid

Miscibility studies revealed that “Transcutol-HP was selected as the co-surfactant”

**Table 2:** NE formulation composition and physical screening studies.

Formulation code	Surfactant : co-surfactant ratio	Oil %	S_mix_%		Water %	Physical Stability test			Inference
			Surfactant	Co-surfactant		Heating	Freeze thaw cycle	Centrifugation	
F1	4:1	10	32	8	50	✓	✓	✓	Passed
F2		20	25	5	50	-	-	-	Failed
F3		18.18	21.82	5.45	54.55	-	-	-	Failed
F4		14.04	16.84	4.21	64.91	-	-	-	Failed
F5		11.94	14.33	3.58	70.15	✓	✓	✓	Passed
F6		14.81	23.71	5.92	55.56	✓	-	-	Failed
F7		11.63	18.61	4.65	65.12	✓	✓	✓	Passed
F8		11.11	19.445	19.445	50	✓	✓	✓	Passed
F9		10	17.5	17.5	55	✓	-	-	Failed
F10		8.89	15.55	15.55	60	✓	-	-	Failed
F11		7.75	13.56	13.56	65.12	✓	-	-	Failed
F12		6.67	11.665	11.665	70	✓	✓	✓	Passed
F13		7.49	18.725	18.725	55	✓	-	-	Failed
F14		6.67	16.665	16.665	60	✓	✓	✓	Passed
F15		5.8	14.495	14.495	65.22	✓	✓	✓	Passed
F16		5	12.5	12.5	70	✓	-	-	Failed

NE formulation composition and physical screening studies, results of which demonstrated that the formulations prepared from the S_mix_ ratio of 4:1 passed the physical stability test.

**Table 3:** Drug loaded NE formulation composition and physical screening studies.

Formulation code	Surfactant : co-surfactant ratio	Oil %	S_mix_%		Water %	Physical Stability test			Inference
						Heating	Freeze thaw cycle	Centrifugation	
F1	4:1	10	32	8	50	✓	✓	✓	Passed
F5		11.94	14.33	3.58	70.15	-	-	-	Failed
F7		11.63	18.61	4.65	65.12	✓	-	-	Failed
F8		11.11	19.445	19.445	50	✓	-	-	Failed
F12		6.67	11.665	11.665	70	✓	-	-	Failed
F14		6.67	16.665	16.665	60	✓	-	-	Failed
F15		5.8	14.495	14.495	65.22	-	-	-	Failed

Composition of drug loaded NE and their physical stability testing, F1 gave a clear and transparent formulation.

**Table 4:** *In-vitro* drug release kinetics of optimized NRG NE.

Various release models	Coefficient of correlation (R^2^)
Zero Order	0.937
First Order	0.861
Higuchi	0.9269
Hixon-crowell	0.899

Data obtained from *in-vitro* drug release studies revealed that highest R^2^ value was obtained in case of Zero order model.

**Table 5:** Pharmacokinetic parameters of the NRG solution (i.v. and i.n.) and NE formulation after i.n. administration.

Formulation	Organ/Tissue	C_max_ (ng/ml)	T_max_ (h)	T_1/2_ (H)	K_e_^(h-1)^	AUC_0-480_ (ng/ml*∗*h)	AUC_0-infinity_ (ng/ml*∗*h)
NRG sol (i.n.)	Brain	870.77 ± 5.4	2	6.84 ± 0.2	0.1 ± 0.003	3352.86 ± 8.9	5813.18 ± 9.2
	Blood	775.44 ± 2.5	1	4.63 ± 0.4	0.14 ± 0.04	1919.734 ± 6.7	2554.17 ± 8.1

NRG NE (i.v.)	Brain	381.67 ± 3.1	1	7.44 ± 0. 8	0.09 ± 0.003	1599.36 ± 4.5	3118.87 ± 11.2
	Blood	2502.74 ± 4.2	0.5	3.13 ± 0.7	0.22 ± 0.007	7533.91 ± 4.5	9582.21 ± 7.8

NRG NE (i.n.)	Brain	1148.64 ± 3.3	2	13.38 ± 1.1	0.05 ± 0.001	5345.13 ± 7.5	13543.23 ± 6.9
	Blood	794.33 ± 7.1	2	10.4 ± 0.3	0.06 ± 0.02	3777.63 ± 5.3	8690.07 ± 8.5

Pharmacokinetic parameters results clearly indicated that the NRG concentration is significantly higher in optimized formulation administered via i.n. (p<0.05) than that of administration of optimized formulation via i.v. and NRG solution via i.v. route.

## Data Availability

All data are provided in full in the results section of this paper.

## Abbreviations

NRG: Naringenin
NE: Nanoemulsion
PD: Parkinson's disease
PDI: Polydispersity index
CNS: Central nervous system
BBB: Blood Brain Barrier
TEM: Transmission electron microscopy
CLSM: Confocal laser scanning fluorescence microscopy
NBM: Narrow beam maze
PMS: Postmitochondrial supernatant
GSH: Glutathione
EDTA: Ethylenediaminetetraacetic acid
TCA: Trichloroacetic acid
TBARS: Thiobarbituric acid-reactive substances
TBA: Thiobarbituric acid
SOD: Superoxide dismutase
LOD: Limit of detection
LOQ: Limit of quantification.
